# Demographic data is more predictive of component size than digital radiographic templating in total knee arthroplasty

**DOI:** 10.1186/s43019-020-00075-y

**Published:** 2020-11-23

**Authors:** Stephen J. Wallace, Michael P. Murphy, Corey J. Schiffman, William J. Hopkinson, Nicholas M. Brown

**Affiliations:** 1 Department of Orthopaedic Surgery and Rehabilitation, Harborview Medical Center, 325 9th Ave, Seattle, WA 98104 USA; 2 Department of Orthopaedic Surgery and Rehabilitation, Loyola University Medical Center, 2160 S. 1st Ave, Maguire Suite 1700, Maywood, IL 60153 USA; 3 Department of Orthopaedic Surgery and Rehabilitation, University of Washington Medical Center, 1959 N.E. Pacific St., Seattle, WA 98195 USA

**Keywords:** Total knee arthroplasty, Multivariate linear regression, Demographic data, Predicting implant size

## Abstract

**Background:**

Preoperative radiographic templating for total knee arthroplasty (TKA) has been shown to be inaccurate. Patient demographic data, such as gender, height, weight, age, and race, may be more predictive of implanted component size in TKA.

**Materials and methods:**

A multivariate linear regression model was designed to predict implanted femoral and tibial component size using demographic data along a consecutive series of 201 patients undergoing index TKA. Traditional, two-dimensional, radiographic templating was compared to demographic-based regression predictions on a prospective 181 consecutive patients undergoing index TKA in their ability to accurately predict intraoperative implanted sizes. Surgeons were blinded of any predictions.

**Results:**

Patient gender, height, weight, age, and ethnicity/race were predictive of implanted TKA component size. The regression model more accurately predicted implanted component size compared to radiographically templated sizes for both the femoral (*P* = 0.04) and tibial (*P* < 0.01) components. The regression model exactly predicted femoral and tibial component sizes in 43.7 and 43.7% of cases, was within one size 90.1 and 95.6% of the time, and was within two sizes in every case. Radiographic templating exactly predicted 35.4 and 36.5% of cases, was within one size 86.2 and 85.1% of the time, and varied up to four sizes for both the femoral and tibial components. The regression model averaged within 0.66 and 0.61 sizes, versus 0.81 and 0.81 sizes for radiographic templating for femoral and tibial components.

**Conclusions:**

A demographic-based regression model was created based on patient-specific demographic data to predict femoral and tibial TKA component sizes. In a prospective patient series, the regression model more accurately and precisely predicted implanted component sizes compared to radiographic templating.

**Level of evidence:**

Prospective cohort, level II.

## Introduction

Preoperative templating for total knee arthroplasty (TKA) attempts to predict component size before implantation. Although radiographs are essential to assess bony alignment, post-traumatic changes, and other potential pathology as part of a preoperative evaluation, radiographic templating for TKA has been shown to be accurate only 40–65% of the time [1–7].

A proper calculation of component sizes has important implications in maintaining a hospital’s implant stock, predicting patient outliers, and streamlining bone preparation and trialing during surgery. As healthcare costs are increasingly being scrutinized, this preoperative information could be used to customize surgical trays, reduce packaging waste, and avoid unnecessary reprocessing.

Recent studies have suggested formulas that predict TKA component size based on demographic data such as gender, height, weight, age, ethnicity/race, and shoe size [8–13]. Although these studies have asserted improved component estimates, the formulas are limited to one implant system or do not directly compare the accuracy of their method to standard templating techniques. Further, it is currently unknown how demographic-based models that predict operatively implanted TKA implant sizes compare with traditional, two-dimensional, radiographic templating methods.

Through a retrospective chart review, a mathematical model using patient-specific demographic data was developed to preoperatively predict component size independent of the implant system. The accuracy of this model was then prospectively compared to standard digital templating in a separate patient cohort.

The aim of this study is to compare the accuracy of TKA component size predictions between routine digital templating to a new mathematical model. To the knowledge of the authors, this is the first study to prospectively compare the accuracy of digital templating to a predictive model based on patient demographic data regardless of implant system.

## Materials and methods

After approval from the Institutional Review Board (IRB), demographic data was retrospectively collected at a single academic institution from a consecutive series of 201 patients (January to December, 2018) undergoing index TKA and used to build a multivariate linear regression model. This multivariate linear regression model used demographic data alone to predict the femoral and tibial component size implanted for that patient. All femoral and tibial component sizes were converted to dimensions of millimeters in the anterior-posterior (AP) dimension and medial-lateral (ML) dimension, according to the published sizes for the respective implant. Demographic data collected included age, height, weight, body mass index (BMI), gender, race, ethnicity, and operative laterality.

Subsequently, 181 consecutive patients were prospectively enrolled to compare traditional, two-dimensional, radiographic templating methods with demographic-based predictions in their ability to accurately predict the femoral and tibial sizes implanted intraoperatively. In total, 382 patients were included in this study.

Surgeons were blinded to both traditional radiographic templating results and demographic-based predictions. Implanted TKA sizes were chosen at the discretion of the operating surgeon according to standard means. Cases requiring revision or conversion TKA were excluded. In cases of bilateral TKA within the study period, only the first operation was included to avoid duplicate demographic input from the same patient.

### Statistical analysis

The four discrete formulas – AP femur, ML femur, AP tibia, ML tibia – were used to prospectively predict component sizes on a patient cohort unique from that on which the regression model was built. The demographic data of 181 unique, prospectively collected patients was input into each regression model resulting in a specific predicted component dimension in millimeters. This measured output was then converted to the corresponding nominal implant size based on published manufacturer specifications for comparison to actual implanted size (Triathlon, Stryker, Kalamazoo, MI, USA and PFC Sigma, DePuy Synthes, Warsaw, IN, USA) By having a regression model that output dimensions in millimeters, as opposed to the specific size of a given implant, the model may be applied to any implant system. Performing surgeons were blinded to the models’ output dimensions and corresponding component size at the time of surgery.

Digital radiographic templating was performed on the same prospective patient cohort using standard preoperative AP and lateral knee radiographs. All radiographs were obtained within 6 months of the TKA surgery. Component size was decided based on “best-fit” methods using the digital templates provided on TraumaCad (Brainlab, Munich, Germany). Specific implant choice was based on attending surgeon preference. For the purposes of this study, radiographic templating was performed by a senior resident (SW) who was blinded of other radiographic templating results and final component sizes implanted. When a calibration marker was not present, a 115% magnification correction was applied. This magnification correction has previously been shown to accurately template arthroplasty component sizes [14–18].

**Fig. 1:**
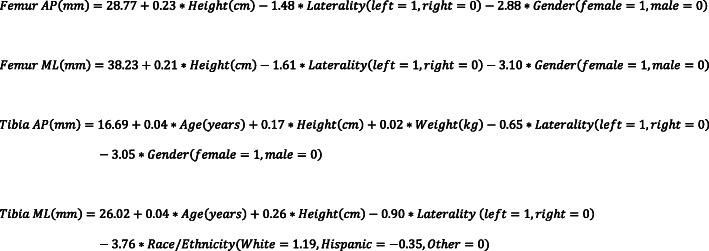
General linear model equations for anterior-posterior (AP) or medial-lateral (ML) dimensions of the femoral or tibial components

An a priori power analysis was first performed to determine the appropriate sample size for this study. To generate a multivariate linear regression model to detect a small effect size in component dimensions (*f*^2^ = 0.05), one would need to enroll 159 subjects to achieve a power of 0.80 and a type I error rate of 0.05. To detect a small effect size in component dimensions (*f*^2^ = 0.2) between a multivariate linear regression model and radiographic templating, one would need to enroll 156 subjects to achieve a power of 0.80 and a type I error rate of 0.05. Four separate general linear regression models were formulated to predict either the AP or ML dimensions of the femoral or tibial components. (Fig. 1). Initial variables included gender, height, weight, age, ethnicity/race, laterality, implant system, and BMI. Table 1 shows the demographic distribution among the retrospective consecutive series of 201 patients and prospective series of 181 patients, totaling 382 patients involved in this study. Using a backward selection procedure, variables with *P* values > 0.05 were removed to improve model parsimony.

**Table 1:** Patient demographics of both the retrospective, multivariate linear regression model group and the prospective group used for comparison of digital templating and the demographic-based sizing equations

Retrospective cohort (***n*** = 201)				Prospective cohort (***n*** = 181)		
*Demographics*		*Number*	*Percentage*	*Demographics*	*Number*	*Percentage*
Gender	Male	73	36.3%	Male	61	33.7%
	Female	128	63.7%	Female	120	66.3%
Laterality	Right	107	53.2%	Right	88	48.6%
	Left	94	46.8%	Left	93	51.4%
Ethnicity/race	White	151	75.1%	White	133	73.5%
	Black	25	12.4%	Black	27	14.9%
	Hispanic	15	7.5%	Hispanic	17	9.4%
	Asian	5	2.5%	Asian	2	1.1%
	Middle Eastern	3	1.5%	Middle Eastern	1	0.6%
	Indian	2	1.0%	Indian	1	0.6%
	*Mean*	*Standard deviation*	*Range*	*Mean*	*Standard deviation*	*Range*
Age (years)	66.0	9.9	42–89	65.8	9.7	44–86
BMI (kg/m^2^)	33.6	7.1	20.2–57.9	34.2	7.1	20.4–62.2
Height (cm)	168.3	10.7	139.7–198.1	167.4	10.7	134.6–193
Weight (kg)	95.3	22.8	53–180.5	96.2	23.1	49.4–181.4

The Wilcoxon signed-rank test and paired Student’s *t* test were used to compare the multivariate linear regression model with routine digital templating in their ability to predict true intraoperative implanted femoral and tibial component size. Statistical significance was set at *P* < 0.05. Statistical analysis was performed using SPSS (IBM, Armonk, NY, USA).

### Source of funding

In preparation of this article, no funding was received in any form whatever.

## Results

The demographic-based multivariate linear regression model conducted on the retrospective consecutive series of 201 patients showed gender, height, weight, age, ethnicity/race were most predictive of implanted component size in TKA (all *P* < 0.05). Laterality, implant model, and BMI were not predictive (all *P* > 0.05). Among the retrospective series, 101 TKAs were Triathlon (Stryker) while 100 were PFC Sigma (DePuy). Among the prospective series, 90 TKAs were Triathlon (Stryker) while 91 TKAs were PFC Sigma (DePuy). Of the four discrete equations, the specific regression model formulas for the AP femoral and ML tibial dimensions were of greatest performance in predicting implanted component size in the prospective series.

**Fig. 2:**
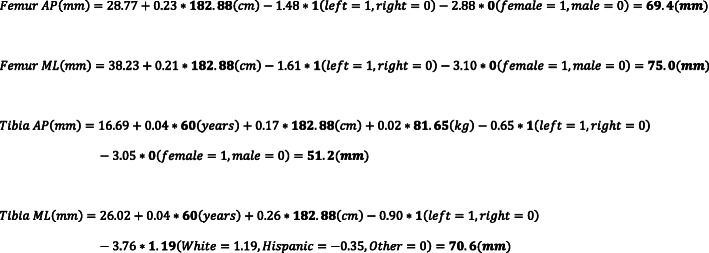
Plot of the digitally templated femoral and tibial sizes versus the sizes calculated by demographic data. Femoral and tibial dimensions in the anterior-posterior dimension (Fig. 2**a** and **c**, respectively) and the medial-lateral dimensions (Fig. 2**b** and **d**, respectively) were subsequently converted to the respective implant size

When used among the prospective series, the demographic-based multivariate linear regression models more accurately predicted implanted component size compared to digital templated sizes for both the femoral (*P* = 0.04) and tibial (*P* < 0.01) components (Fig. 2). The regression models exactly predicted the femoral component in 79 (43.7%) out of 181 prospective cases and the tibial component in 79 (43.7%) cases. Radiographic templating matched the femur and tibia in 64 (35.4%) and 66 (36.5%) cases, respectively. The demographic-based regression models were predictive within one size of the implanted femoral and tibial components in 163 (90.1%) and 173 (95.6%) of cases, compared to 156 (86.2%) and 154 (85.1%) with digital templating. The demographic-based regression models predicted the femoral and tibial components within two sizes in every case. Radiographic templated sizes varied up to four sizes for both the femoral and tibial components (Tables 2 and 3). The demographic-based regression models averaged 0.66 and 0.61 sizes from the implanted femoral and tibial components compared to 0.81 and 0.81 sizes for the templated predictions, respectively.

**Table 2:** Number of times the digitally templated (columns) and demographically calculated (rows) predicted an exact match (“Perfect”), within 1 size (“Close”), and > 1 size (“Off”) from the implanted total knee arthroplasty component. The demographically calculated size was first calculated according to femoral anterior-posterior (AP), femoral medial-lateral (ML), tibial anterior-posterior, and tibial medial-lateral dimensions (in descending order) and subsequently converted to the respective implant size

Femoral AP dimension				
		Templated		
		Perfect (0)	Close (1)	Off (≥ 2)
Calculated	Perfect (0)	33	35	11
	Close (1)	26	46	12
	Off (2)	5	11	2
Femoral ML dimension				
		Templated		
		Perfect (0)	Close (1)	Off (≥ 2)
Calculated	Perfect (0)	32	32	11
	Close (1)	27	50	13
	Off (2)	5	10	1
Tibial AP dimension				
		Templated		
		Perfect (0)	Close (1)	Off (≥ 2)
Calculated	Perfect (0)	26	41	12
	Close (1)	36	44	15
	Off (2)	4	3	0
Tibial ML dimension				
		Templated		
		Perfect (0)	Close (1)	Off (≥ 2)
Calculated	Perfect (0)	27	40	12
	Close (1)	34	45	15
	Off (2)	5	3	0

**Table 3:** Number and frequency the demographically calculated and digitally templated sizes differed from the sizes implanted. Calc FemAP represents the demographically calculated size from the femur in the anterior-posterior dimension, while Calc TibAP represents that for the tibia. Calc FemML represents the demographically calculated size from the tibia in the medial-lateral dimension, while Calc TibML represents that for the tibia

Sizes away from implanted	Exact match (0)	± 1	± 2	± 3	± 4
Templated femur	64 (35.36%)	92 (50.83%)	21 (11.6%)	3 (1.66%)	1 (0.55%)
Templated tibia	66 (36.46%)	88 (48.62%)	23 (12.71%)	3 (1.66%)	1 (0.55%)
Calc FemAP	79 (43.65%)	84 (46.41%)	18 (9.94%)	0 (0%)	0 (0%)
Calc FemML	75 (41.44%)	90 (49.72%)	16 (8.84%)	0 (0%)	0 (0%)
Calc TibAP	79 (43.65%)	95 (52.49%)	7 (3.87%)	0 (0%)	0 (0%)
Calc TibML	79 (43.65%)	94 (51.93%)	8 (4.42%)	0 (0%)	0 (0%)

## Discussion

This study demonstrates that demographic data may reliably predict femoral and tibial component dimensions. Specifically, the above multivariate linear regression model supports the notion that the AP femoral and ML tibial dimensions are superior in predicting implant size than radiographic digital templating. Because the model produces a measured output dimension in millimeters, it can be converted to any implant system with knowledge of the manufacturers’ implant specifications.

**Fig. 3:**
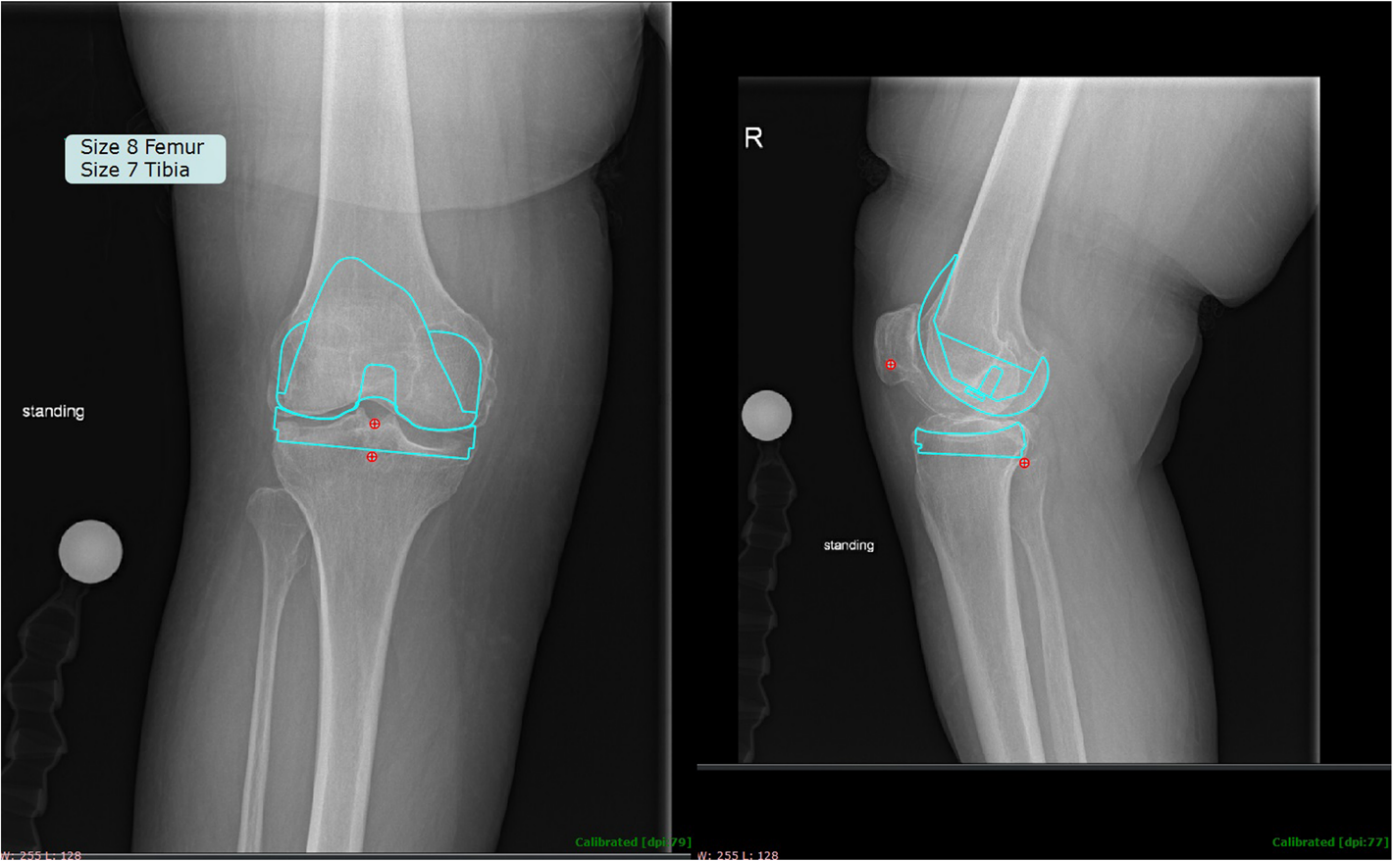
Example case where demographic information outperformed radiographic templating. Here the patient’s demographic information calculated a size-4 and size-3 femoral and tibial component, respectively; perfectly matching the sizes implanted. Meanwhile, radiographic templating showed good fit with a size-8 and size-7 femoral and tibial component, respectively

**Fig. 4:**
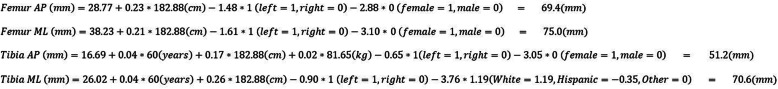
Example calculation of implant sizes for a patient (white male, left-sided, 6 ft/182.88 cm tall, weighing 180 lb/81.65 kg, age 60 years). These dimensions would then be used to identify the closest matching component size for a given implant model

Using demographic data to predict implant sizes was both more accurate and more precise when compared to radiographic templating techniques. The multivariate linear regression model predicted within one size of the implanted femoral and tibial components 90.1 and 95.6% of the time, compared to 86.2 and 85.1% with radiographic templating, respectively. The multivariate linear regression model predicted within two sizes of the implanted femoral and tibial components in every case, compared to within four sizes for radiographic templating, respectively. Similarly, the multivariate linear regression model averaged within 0.66 and 0.61 sizes from the implanted femoral and tibial components, compared to within 0.81 and 0.81 for radiographic templating, respectively. The results demonstrate that the use of demographic data in predicting femoral and tibial implant size outperformed radiographic templating on every measure. An example where demographic data perfectly matched that implanted, while radiographic templating was off by four sizes for both the femoral and tibial components, respectively, is shown in Fig. 3. Similarly, an example calculation of how the demographic data may be used to predict component sizes is shown in Fig. 4.

Radiographs provide essential information for diagnosis and for anticipating surgical prerequisites. Knowledge of a patient’s unique anatomy or pathologic changes can facilitate a more streamlined surgery. Traditionally, radiographs have also been utilized to predict component size in total knee and hip arthroplasty. Despite the widespread use of digital radiographs and digital software, radiographic templating has shown variable accuracy in predicting TKA component size [19–21]. Meanwhile, the concept of maximizing value in healthcare has grown with several studies examining methods of more efficient and affordable delivery of patient care [21–23].

Recently, several studies have been published that support predicting TKA component size based on demographic data. All authors report *R*^2^ values ranging 0.50 to 0.79 and accuracy within one size in 85% to 100% of cases [8–11, 24]. Recently, the equations presented by Bhowmik-Stoker et al. were shown to be most accurate when applied to a unique patient population, still averaging within one size 88 and 92% of the time for femoral and tibial sizes, respectively [25]. The results of this study substantiate these authors’ sentiments, that demographic data may be more accurate and precise than the current standard.

The data presented here is not meant to suggest that there is no role for radiographic templating. TKA templating based on demographic variables alone will not account for deformity correction, bone loss, anatomic variants, or other factors that may be better accounted for with traditional techniques. Rather, for surgeons frequently using templating resources, this study supports a statistically superior method to validate their results and coordinate their surgical resources. With reliable predictions of implant sizes, operating room efficiency may be streamlined and the surgical team may be better prepared for potential outliers.

This study has several limitations. The study involved the implantation of only two total knee implant systems. In an effort to standardize the results and reduce implant-related sizing bias, the multivariate linear regression model was designed to predict femoral and tibial dimensions in millimeters. These dimensions were subsequently converted to the respective implant size of the surgeon’s choosing. Further, the two implant designs were included in the statistical analysis and were not found to be statistically significant in determining implant size. However, there still exists the potential that the results may slightly vary for other implant systems.

Additionally, this study evaluated the predicted component size to the reported component implanted during surgery. This assumes that the implanted component size was ideal for the patient. No post-operative measures were assessed to gauge whether implants were oversized or undersized.

Another limitation involves the use of patients from a single institution. The patient population from a single institution may not fully encompass that of another institution, practice, or country in general. However, a recent study assessed the generalizability of several other predictive formulas in a separate population [25]. This study identified only minor differences in accuracy, ranging from 85 to 100% in sizing accuracy in the original published work, compared to 79–92% in a separate population [8–11, 24, 25]. Similarly, the cohort used to design the equations used in this study was intentionally omitted from the assessment comparing demographic versus digital templating. Rather, a prospectively collected cohort was used for the final comparison.

A final limitation is that 53.6% (97/181) of radiographs used for templating in the prospectively collected patients did not have calibration markers. This could have affected radiographically templated sizes. The authors chose not to eliminate subjects without appropriately placed calibration markers given that this may bias the results and inappropriately represent standard practice. Further, even with a calibration marker present, proper marker placement is difficult to confirm for accurate calibration and measurement. In cases without calibration markers, digital radiographic templating was performed at a magnification correction of 115% given its previously published accuracy in templating component sizes [14–18]. For the results of this study to be applied more broadly, it was believed that current radiographic techniques should not be modified.

## Conclusions

This study retrospectively designed a simple, multivariate linear regression model based on patient demographic data that may predict TKA component size. Subsequent testing on a prospective patient cohort showed that these formulas had statistically superior accuracy and precision when compared to traditional digital templating techniques. These equations may provide an improved preoperative plan for patients undergoing TKA.

## Data Availability

The authors are happy to provide data upon request.
